# A Rare and Unusual Case of Hypernatremic Dehydration in a Newborn Presenting With Adrenal Haemorrhage and Leading to Acute Kidney Injury

**DOI:** 10.7759/cureus.61265

**Published:** 2024-05-28

**Authors:** Mahaveer S Lakra, Bhavana B Lakhkar, Amar Taksande, Sagar Karotkar, Ashwini Lakra, Mayur B Wanjari, Roshan Prasad

**Affiliations:** 1 Pediatrics, Datta Meghe Medical College, Datta Meghe Institute of Higher Education and Research, Nagpur, IND; 2 Pediatrics, Jawaharlal Nehru Medical College, Datta Meghe Institute of Higher Education and Research, Wardha, IND; 3 Research and Development, Jawaharlal Nehru Medical College, Datta Meghe Institute of Higher Education and Research, Wardha, IND

**Keywords:** adrenal haemorrhage, jaundice, neonate, abdominal lump, acute kidney injury

## Abstract

Adrenal haemorrhage, although a rare entity in the neonatal period, is a known complication of birth asphyxia. Adrenal haemorrhage progresses differently depending on the type and extent of the glands involved. Adrenal haemorrhage can cause persistent jaundice, fever, dehydration, scrotal swelling, abdominal wall discolouration, septicemia, and a shock-like state. Here, we report the case of a four-day-old male infant who presented with jaundice, poor feeding, and hypernatremic dehydration. The patient developed acute kidney injury and, eventually, renal failure due to adrenal haemorrhage. He had an abdominal lump with deranged renal parameters along with hyperbilirubinemia. Abdominal ultrasonography and contrast computed tomography scan showed left suprarenal enlargement with evidence of adrenal haemorrhage. The patient was managed well with ventilatory support and peritoneal dialysis and discharged successfully. A subsequent follow-up showed complete resolution of the adrenal haemorrhage. Single ultrasonography is a good modality for diagnosis but not sufficient, so serial ultrasonography at subsequent follow-up is a must.

## Introduction

Adrenal haemorrhage is a rare entity seen in the neonatal period and may have a devastating course. The presentation and manifestation of adrenal haemorrhage may vary depending upon the type, severity, and organ involved. It may be unilateral or bilateral, cortical or subcapsular, depending upon the aetiology and extent of involvement of the haemorrhage [1]. It is mostly seen after a complicated and difficult delivery, acute trauma, hypovolemia, or metabolic acidosis. Difficult deliveries, acidosis, and large babies who have undergone birth asphyxia are more prone to adrenal haemorrhage. Lethargy, dehydration, feed intolerance, jaundice, hypertension, dehydration, scrotal swelling, and temperature instabilities may be the various presentations. It can range from asymptomatic to a shock-like state that manifests as an emergency catastrophic event such as an adrenal crisis [2]. Here, we are reporting the case of a newborn who presented with hypernatremia and dehydration with adrenal haemorrhage leading to acute kidney injury and ultimately renal failure, which is a rare phenomenon.

## Case presentation

A four-day-old, full-term male neonate weighing 3.1 kg was born to a primigravida mother and presented with increased respiratory rate, yellowish discolouration of the skin, and decreased urine output. The mother had regular antenatal visits, and her period was uneventful. The child was born by spontaneous vaginal delivery after nine hours of labour and four hours of membrane rupture. He cried immediately after birth; the one- and five-minute APGAR scores were 7 and 9, respectively. The history was otherwise unremarkable.

The patient was conscious and lethargic on physical examination, with laboured breathing and signs of dehydration. The vital parameters were 112 beats per minute, 88 cycles per minute, temperature of 36.7°C, saturation of peripheral oxygen (SpO2) of 92%, and a delayed capillary refill time. He had no pallor, icterus was present involving the face and upper trunk (Kramer stage 2), and all primitive reflexes were elicitable. All peripheral pulses were well felt; blood pressure was 54/32 mmHg with a Downes score of 3 (respiratory rate (RR) > 80, mild subcostal retractions, and nasal flaring). On bimanual examination, the abdomen was flat, with a palpable mass in the left lumbar region, with no other organomegaly and no tenderness, bruise, skin discolouration, or sign of fluid collection. Both testicles were palpable in the scrotum, and there was no scrotal swelling or discolouration. The respiratory and cardiovascular systems were normal.

The patient was investigated with an initial diagnosis of full term, appropriate for gestational age, and physiological jaundice with late-onset sepsis, and laboratory investigations were done, the results of which are mentioned in Table 1.

**Table 1:** Laboratory reports of the patient and normal values. WBC: white blood cell; CRP: c-reactive protein

Investigation	Results	Normal value
Hematocrit (%)	65.1	36-47
Total WBC count (/mm³)	20600	4000-13500
Total platelet count (lakh/µL)	4.12	1.5-4.5
CRP (mg/L)	15.11	2-5
Urea (mg/dL)	398	5-20
Creatinine (mg/dL)	14.4	0.5-1
Total bilirubin (mg/dL)	18.6	<1.5
Sodium (mEq/L)	188	130-147
Potassium (mEq/L)	5.2	3.5-5.1
Serum cortisol (µg/dl)	8.4	5-25

**Figure 1:**
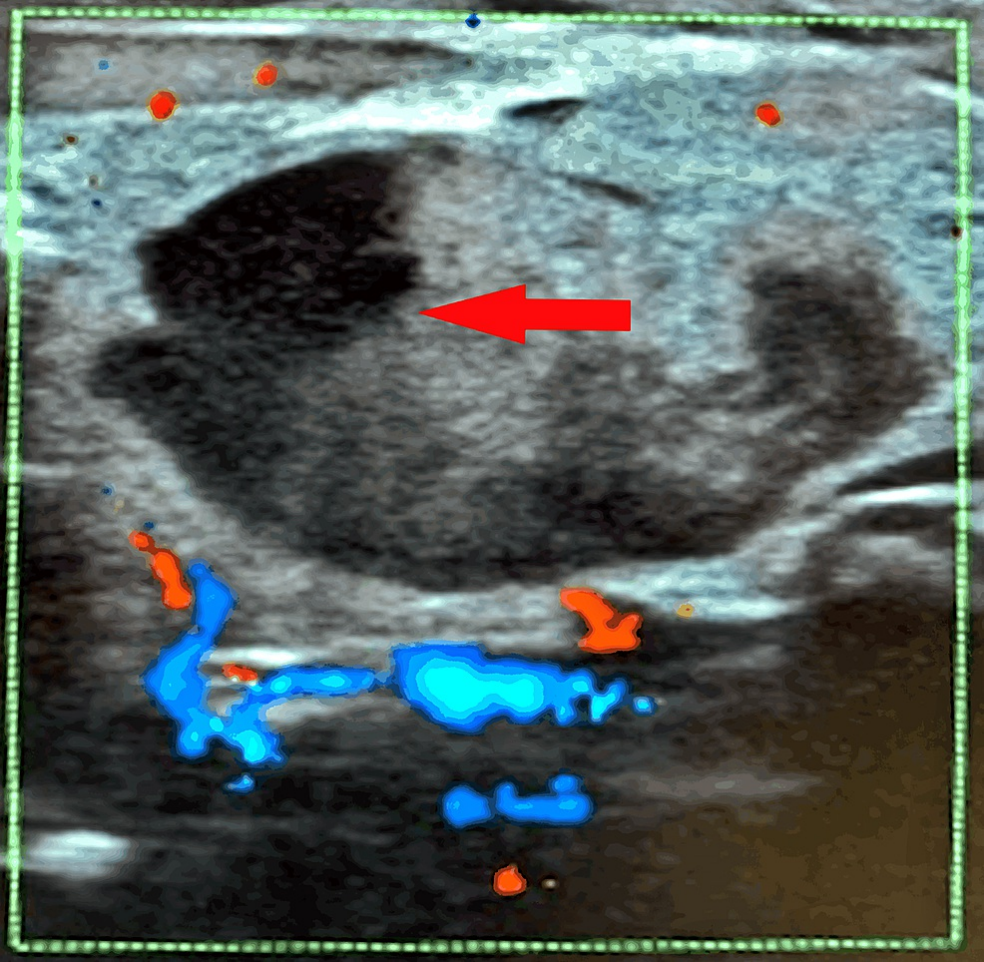
Heteroechoic lesion in left suprarenal gland

The arterial blood gas suggested metabolic acidosis, which required a normal saline bolus and soda bicarbonate infusion. Abdominal ultrasound was ordered due to a palpable mass, which revealed a well-defined heteroechoic lesion in the left suprarenal region, medium-level floaters within the lesion, and no significant vascularity or hyperemia, causing compression of the upper pole of the left kidney, as shown in Figure 1.

**Figure 2:**
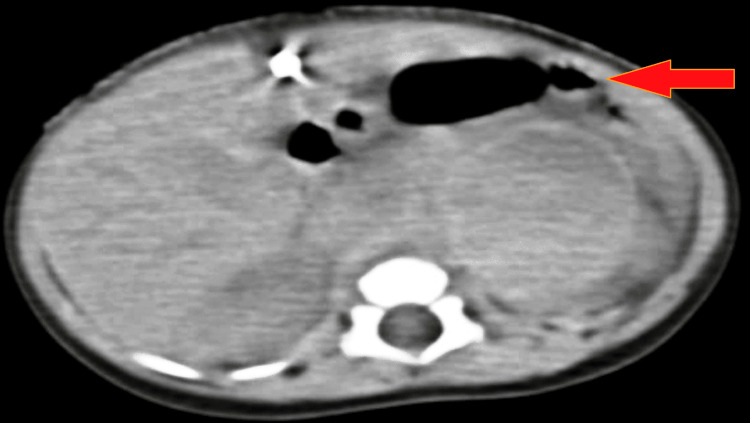
CT Scan of adrenal gland reveals left supra renal haemorrhage as shown by arrow

Abdominal CT scan revealed solid, well-defined, hypo-dense, non-enhancing, fluid-attenuated left suprarenal gland masses (2.6 cm by 2.4 cm) with a conclusion of left suprarenal gland collection, likely haemorrhage, as shown in Figure 2. The neonate and mother underwent a chest x-ray, blood culture, and blood grouping.

The neonate was admitted to the neonatal intensive care unit (NICU), and double surface phototherapy, oxygen support by nasal prongs, and IV fluids were started. The dehydration and renal failure were managed as per the standard guidelines. The treatment for hyperkalemia and renal failure was started, and the baby was managed conservatively. The cortisol level was sent, which was normal, so the baby did not require an injection of hydrocortisone. At a subsequent follow-up, the child was doing well and the adrenal swelling subsided over the next month.

## Discussion

Adrenal haemorrhage, though a rare presentation in newborns, can be encountered in routine practice in NICUs and is mostly seen in the first week of life. The aetiology of adrenal haemorrhage varies, and chances are higher in term babies because of factors such as large size, instrumental delivery, macrosomia, prolonged, complicated delivery, coagulopathy, and birth trauma [3]. Two similar cases were reported by Alabsi et al., where the first baby presented with scrotal discolouration but the second baby presented with oliguria and hypertension with renal failure [4]. In the first case, the aetiology was renal venous thrombosis due to oliguria. In the present case, although the patient presented with hypernatremic dehydration with oligouria, renal venous thrombosis was not seen.

Adrenal haemorrhage symptoms can range from asymptomatic to life-threatening and devastating adrenal involvement leading to shock. It may present with evidence of birth asphyxia, abdominal swelling, scrotal discolouration, jaundice, and metabolic or electrolyte disturbances. [5]. Because the involvement is sometimes subcapsular and the cortex is occasionally spared, these patients rarely experience an adrenal crisis because the haemorrhage is mostly subcapsular. Adrenal insufficiency is not seen unless 90% of the gland is involved [5,6]. Sometimes cortisol levels are affected, and patients may get adrenal crises. The relatively large size of the adrenal gland may be a risk factor in neonates, who are more susceptible to injuries. Other risk factors may be breach presentation, instrumental delivery, acidosis, and macrosomia [5-7]. Because of the location of the haemorrhage between the liver and the spine, 70% of the adrenal haemorrhage is seen on the right side. Another reason is that the right adrenal vein drains directly into the inferior vena cava, making it more prone to compression, resulting in venous congestion and a significant change in pressure. The type of haemorrhage, in this case, was on the left side in contrast to the right, as reported by Ruminiska et al. [8] and Demirel et al. [9]. They reported the predominance of right-sided haemorrhage compared to the left side [8,9]. Ninety percent of haemorrhages are unilateral, and 75% occur on the right [7]. Singh et al. reported a case of adrenal haemorrhage with jaundice [10], similar to the present case. In a report by Tognato et al., birth asphyxia was the cause of adrenal haemorrhage in two neonates, but no aetiology was found in one [11]. Wright et al. described a newborn with an abdominal lump diagnosed as an adrenal haemorrhage [3].

Abdominal trauma, neuroblastoma, and Wilms tumour should all be considered differential diagnoses. Neuroblastoma and Wilms' tumour should be ruled out if a flank mass is palpable. Neuroblastoma should be ruled out if the size doesn’t regress to normal at a subsequent follow-up [7]. Ultrasonography is the most essential early diagnostic test, but MRI contrast is the most diagnostic and confirmatory test. Abdominal ultrasonography might pick up prenatal adrenal haemorrhage if done carefully. Ideally, follow-up should be done for six months, and at least until 90 days, as maximum resolution occurs within 60 days [8].

The treatment modalities mainly depend on the presentation and severity of the illness. The patient may be asymptomatic or have a more complicated presentation, such as an adrenal crisis with sudden deterioration and a shock-like state. If the child is hemodynamically stable, then a close watch is required. Hydrocortisone therapy may be required if the patient shows electrolyte derangement with a shock-like presentation [11]. Patients with adrenal crisis and severe adrenal haemorrhage may require hydrocortisone with or without fludrocortisone. Following ultrasonography visits, the patient should be monitored for adrenal size and haemorrhage regression. In our case, the patient responded well to the treatment, and the lump resolved over time.

## Conclusions

Adrenal haemorrhage, though a rare entity in neonatal life, can have a varied presentation and complicate an underlying neonatal illness. Unusual and non-specific presentations of adrenal haemorrhage may be seen in neonates, and sometimes it can be picked up by clinical examination or ultrasonography of the abdomen as an incidental finding. Babies may be asymptomatic or may present with jaundice, oliguria, an abdominal lump, and other symptoms of catastrophic adrenal insufficiency. Conservative management and proper follow-up are the keys to management.
